# Estimating the emergency care workforce in South Africa

**DOI:** 10.4102/phcfm.v13i1.3174

**Published:** 2021-12-08

**Authors:** Ritika Tiwari, Raveen Naidoo, René English, Usuf Chikte

**Affiliations:** 1 Department of Global Health, Faculty of Health and Medical Sciences, Stellenbosch University, Cape Town, South Africa; 2 Emergency Medical Services and Disaster Medicine, National Department of Health, Pretoria, South Africa

**Keywords:** emergency care personnel, paramedics, health policy, health workforce forecasting, health systems, health service strengthening, South Africa; health worker

## Abstract

**Background:**

Emergency care is viewed as a fundamental human right in South Africa’s constitution. In the public sector, all emergency medical services (EMS) come under the Directorate: Emergency Medical Services and Disaster Medicine at the National Department of Health (NDoH), which provides regulation, policy and oversight guidance to provincial structures.

**Aim:**

The aim of the study is to understand the supply and status of human resources for EMS in South Africa.

**Setting:**

This research was undertaken for South Africa using the Health Professions Council of South Africa (HPCSA) database from 2002 to 2019.

**Methods:**

A retrospective record-based review of the HPCSA database was undertaken to estimate the current registered and future need for emergency care personnel forecasted up to 2030.

**Results:**

There are 76% Basic Ambulance Assistants registered with HPCSA. An additional 96 000 personnel will be required in 2030 to maintain the current ratio of 95.9 registered emergency care personnel per 100 000 population. The profile of an emergency care personnel employed in South Africa is likely to be a black male in the age group of 30–39-years, residing in one of the economically better-resourced provinces.

**Conclusion:**

It is time that the current educational framework is revised. Policy interventions must be undertaken to avoid future shortages of the trained emergency care personnel within South Africa.

## Introduction

Across the world, emergency conditions constitute a large part of the global burden of disease.^1,2,3^ As the world population grows in numbers and age, there is an complementary increase in the demand for acute curative services response to life-threatening emergencies, acute exacerbation of chronic illnesses and several routine health problems that still require prompt action.^4^ High-quality emergency care is seen as a potential tool to address and prevent a substantial portion of death and disability worldwide.^4,5,6^ Thus, emergency care interventions and services must be integrated with primary care and public health measures to ensure comprehensive strengthening of health systems.^4^

Pre-hospital emergency care broadly has three components: Care in the community, care during transportation, which is related to access and care on arrival at the receiving health facility.^7^ Emergency care is designed to overcome the factors most commonly implicated with preventable mortality, such as postponements in seeking care, access to a health facility and the provision of adequate care at the facility.^7^ For lower-middle-income countries (LMICs), it has been suggested that priority be placed on developing minimum guidelines for emergency care that may save lives (and at what cost?)^3^

Within South Africa, emergency care is viewed as a fundamental human right.^8^ There is no clarity about the exact meaning of emergency care being a human right. There is no explanation about whether all persons only have the right to basic emergency care or only definitive and intensive care treatment.^9^ All emergency medical services (EMS) in public and private sector are under the purview of Directorate: EMS and Disaster Medicine at the National Department of Health (NDoH) in Pretoria, with regulation, policy and oversight guidance provided to provincial structures.^9^

Emergency care services are delivered in South Africa at two levels of care pre-hospital sector that has public and private arenas, with nationally based private services and provincial public services^9^ and hospital-based care that has been traditionally staffed and managed for basic, intermediate and advanced life support and emergency care.^10^

Emergency care delivery is an essential part of health systems. To deal with the increasing burden of diseases, a robust health system is needed as it can help translate healthcare services into improved health outcomes. Health systems across the globe are dependent on the health workforce in improving health outcomes. Within Africa, there are limited healthcare resources and critical shortages of trained healthcare personnel in all cadres.^11^

South Africa has a quadruple burden of disease because of communicable diseases such as human immunodeficiency virus/acquired immunodeficiency syndrome (HIV/AIDS) and tuberculosis (TB), maternal and child mortality, non-communicable diseases such as hypertension and cardiovascular diseases, diabetes, cancer, mental illnesses and chronic lung diseases such as asthma and injury and trauma,^12^ and continues to struggle with control of these long-standing national health concerns. As per the cause of death profile for South Africa for the period 1997–2012; HIV/AIDS and TB constituted 33.6% of total deaths followed by cardiovascular diseases 18.5%, injuries 9.6% and cancers 8.7%.^13^ Complications arising from these diseases contribute to the need for appropriate emergency and critical care services.

However, the burden of these diseases and the subsequent need for emergency and critical care services is excessive; the available infrastructure, associated human resources, diagnostic and treatment capabilities, and financial resources devoted to the provision of emergency and critical cares services are not optimum. Despite these challenges, patients with illnesses and injuries are provided emergency and critical care services at the district and regional hospital levels^14^ and in remote rural areas.^14,15^ However, rural district hospitals lack trauma surgical services and other medical specialities, consistent with district hospitals in different countries across sub-Saharan Africa.^16,17,18^

Over the past decade, there have been some changes in emergency care education in South Africa. The Health Professions Council of South Africa (HPCSA) registrations for the following four categories have been closed: Basic Ambulance Assistant (BAA), Ambulance Emergency Assistant (AEA), Emergency Care Technician (ECT) and Paramedic (ANT). For ECT, the old 2-year ECT qualification has been stopped and replaced with a Diploma in Emergency Medical Care that now leads to registration as a ANT. For ANT, the short course Critical Care Assistant (CCA) and 3-year diploma have also been stopped, therefore the registrations have dropped over the past year, but registrations are expected to pick up as the 2-year diploma graduate training ramps up. For AEA, the short course has been replaced by a 1-year Higher Certificate in Emergency Medical Care.

Currently, a barrier exists towards accessing higher education for many of the EMS staff.^19^ Also, the articulation between the short courses and the higher education offerings becomes increasingly complex. The knowledge gap between non-credit bearing short courses and the higher education qualifications grows wider.^19^ Thus, for career pathing and aligning formal qualifications to National Qualifications Framework (NQF), the Higher Education Qualifications Framework (HEQF) has implemented a restructured framework of education.^20^ Two implicit progression routes have been determined: the academic route from Bachelor’s degree, Honours to Master’s and Doctoral study and the professional route from Diploma to Advanced Diploma to Postgraduate Diploma to Master’s and then Doctoral studies.^21^ A similar revision has been carried out in the National Emergency Care Education and Training Policy, focusing on training for cadres in EMS to be offered at three levels – a 1-year higher certificate, 2-year diploma and 4-year bachelor’s degree. Thus, mid-professional level (post graduate entry level - NQF 8) entry would be at master (NQF 9) and doctoral level (up to NQF 10).

Research into existing human resources in emergency care, particularly in South Africa, is needed to understand the scope of shortages and further juxtapose this gap as to what would constitute a core package for emergency care services. Thus, this study aims to report the current HPCSA registered personnel. The main objectives of this include describing the demographic trends of emergency care personnel registered with the HPCSA from 2002 to 2019 and forecasting the need for emergency care personnel up to 2030 for South Africa. This study is a first step towards understanding the supply and status of human resources for EMS in South Africa.

## Methods

Profiling the demographic characteristics of the Emergency Care Professionals.

### Study design

This was a retrospective record-based review of the HPCSA database from 2002 until 2019.

### Setting, study population and sampling strategy

The database was procured by the Department of Global Health, Stellenbosch University, through a special written request made to the HPCSA. The complete database (census) was considered for the analysis that included data on all emergency care personnel under the following categories: AEA, BAA, Emergency Care Practitioner (ECP), ECT and ANT including age, sex, population group and location. In this article, we have used the term population group in line with the definitions in the *Population Registration Act (Act No. 30 of 1950),*^22^, which previously classified South African citizens into four major population categories: White people, ‘coloured’ people (mixed ancestry) ‘Indian people’ and ‘Black people’.^22^ Although the legislation was repealed in 1991, the population categories are still used in reporting in sectors such as the Department of Higher Education. Racial data remain essential in monitoring the redress in the education and training of emergency care personnel who were previously denied access to such training in terms of legislation.

The emergency care personnel categories, along with their educational courses (aligned with NQF levels) and the date of registrations closed for new registrations have been provided in Table 1.

**TABLE 1:** Categories and courses of emergency care professionals.

Emergency care personnel category^19^	Name of course^19^	Type of course^19^	Alignment with NQF level and credits^19^	Date register closed for new registrations/last offering^23^	Scope of practice^24^
BAA	BAA	4-week short course	Not aligned to NQF	January 2018	BLS
AEA	AEA	3-month short course	Not aligned to NQF	January 2020	ILS
ANT	CCA	9-month short course	Not aligned to NQF	January 2018	ALS
	National Diploma: Emergency Medical Care	3-year qualification	NQF 7; 360 credits	January 2020	
ECT	National Certificate: Emergency Care	2-year qualification	NQF 5; 240 credits	Last offering 2019	ALS
ECP	B Tech: Emergency Medical Care	1-year qualification	NQF 7; 120 credits	Last offering 2019	ALS
	Bachelors Degree: Emergency Medical Care	4-year qualification	NQF 8; 480 credits	Registration ongoing	

AEA, Ambulance Emergency Assistant; ALS, Advanced Life Support; ANT, Paramedic; BAA, Basic Ambulance Assistant; BLS, Basic Life Support; CCA, Critical Care Assistant; ECP, Emergency Care Practitioner; ECT, Emergency Care Technician; ILS, Intermediate Life Support; National Qualifications Framework.

### Data collection and data analysis

The relevant de-identified data were collected for the mentioned categories listed on the database using a standardised data collection sheet. This included numbers registered each year, numbers and distribution by healthcare sector (including public/private) and province, qualifications and experience and the demographic profile by sex, population group and age. A similar approach was adopted in previous studies^25,26^ in which relevant data was collected using a data collection sheet that included the following variables: (1) category of health personnel (emergency care personnel), (2) geographical location, (3) age groups, (4) population group and (5) sex. Data were entered into a Microsoft Excel spreadsheet and analysed using the Statistical Package for the Social Sciences (SPSS version 22.0). Frequency distributions, cross-tabulations and graphical representations were used as descriptive statistical methods. Anonymity and confidentiality of all personnel were ensured as the data accessed from the HPCSA and presented in this article is de-identified. In addition, the personnel and salaries management system (PERSAL) data for 2019^27^ was also compared with HPCSA registration data for estimating the privatisation of emergency care professionals.

### Estimating and forecasting the need for emergency care professionals

#### Study design

The forecasting estimates for the need for emergency care professionals were undertaken from 2020 to 2030.

**Setting, study population and sampling strategy:** A forecasting exercise was undertaken for all five emergency care professionals’ categories using the HPCSA database for the entire workforce (census).

**Data collection and data analysis:** South Africa’s population projections from 2020 up to 2030 were estimated from the population estimates and projections as per the World Bank.^28^ The annual supply of emergency care personnel was estimated from the HPCSA database. The unique registrations carried out during the year 2019 under the registry names ‘Ambulance Emergency Assistant’, ‘Basic Ambulance Assistant’, ‘Emergency Care Practitioner’, ‘Emergency Care Technician’ and ‘Paramedic’ were considered as the number of trained personnel available for the South African health system in May 2019.

The attrition from the workforce pool was calculated from the year 2020 to 2030 occurring in the form of retirement (at the age of 65 years) using the HPCSA registration database. The supply was calculated on the basis of unique registrations carried out in all five categories from 2014 to 2019. The future supply was forecasted from 2020 to 2030 using the exponential smoothing technique.

Net emergency care workforce for 2020 was calculated using the following formula:

Net workforce (2020) = Number of personnel (registered as per HPCSA 2019 database) + Forecasted Supply (2020) – Retired personnel (as per HPCSA 2019 database) [Eqn 1]

For subsequent years, a net workforce of previous year (*n*) was assumed to be equivalent to number of personnel for the next year (*n*+1). Whereas forecasted supply and exits through retirements were different for each year.

The current number of emergency care personnel per 100 000 population was calculated for the year 2020 and the same was used as the target ratio up to 2030. Thus, the requirement for number of emergency care professionals to maintain current (2020) status quo up to 2030 was forecasted.

### Ethical considerations

Ethical approval and a request for waiver of informed consent for this retrospective study was obtained from the Stellenbosch University Health Research Ethics Committee (HREC reference number: X19/06/016).

## Results

### The demographic profile of emergency care personnel

Workforce composition: In 2019, there were a total of 56 894 professionals who constituted the overall emergency care personnel registered with HPCSA. Of these, 10 545 AEA, 43 045 BAA, 705 ECP, 1123 ECT and 1476 ANT were recorded in the database until May 2019.

### Profile of emergency care personnel

**FIGURE 1:**
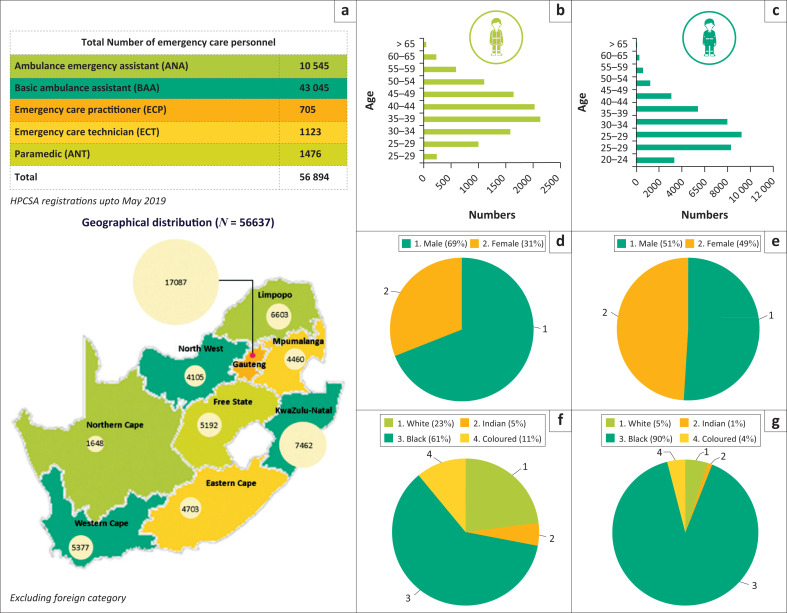

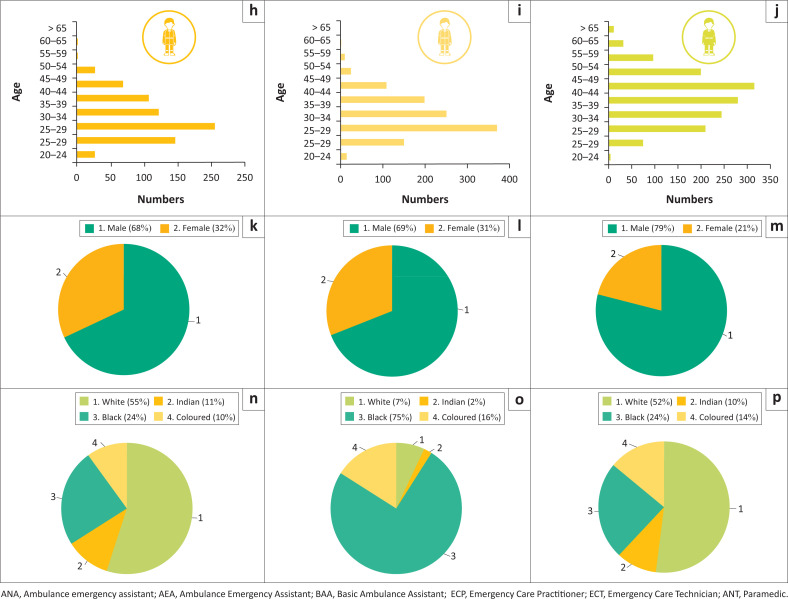
Demographic profile of emergency care personnel in South Africa (*n* = 56 894). (a) Geographical distribution (*N* = 56 637), (b) AEA (*N* = 10 545), (c) BAA (*N* = 43045), (d and e) Sex and (f and g) populations group - Demographic profile for AEA and BAA categories. Demographic profile of emergency care personnel in South Africa (*n* = 56 894). (h) ECP (*N* = 705), (i) ECT (*N* = 1123), (j) ANT (*N* = 1476), (k, l and m) sex, (n, o and p) population group - demographic profile for ECP, ECT and ANT categories.

Figures 1 provide a summary of the data according to geographical distribution, provincial distribution, age, sex and population group. Each of these will then be discussed in more detail. The average age of professionals registered under various emergency care categories was observed to be: AEA 41 years, BAA 35 years, ECP 35 years, ECT 36 years and ANT 43 years. In addition, when the HPCSA registrations are compared with the PERSAL numbers^27^ – approximately 23% Ambulance and related workers (AEA & BAA) and 69% Emergency services related worker (ECP, ECT & ANT) were estimated to be working within the public sector.

Geographical distribution: As per the HPCSA database (in 2019), the majority resided in Gauteng (17 087; 30.03%) followed by the KwaZulu-Natal (KZN (7462; 13.12%) as shown in Table 2.^28^ Over 60% AEA resided in Gauteng, KZN and Western Cape (WC). Similarly, 49% BAA, 89% ECP, 57% ECT and 76.4% ANT resided in these three provinces collectively. The Northern Cape had the lowest number (2.9%) of personnel who were registered there.

**TABLE 2:** Geographical distribution of emergency care personnel (*N* = 56 894 [100%]).

Provinces	AEA		BAA		ECP		ECT		ANT		Total	
	*N*	%	*N*	%	*N*	%	*N*	%	*N*	%	*N*	%
Gauteng	2657	25.20	13 421	31.18	201	28.51	302	26.89	506	34.28	17 087	30.03
KwaZulu-Natal	2017	19.13	4923	11.44	167	23.69	75	6.68	280	18.97	7462	13.12
Mpumalanga	678	6.43	3691	8.57	10	1.42	35	3.12	46	3.12	4460	7.84
Western Cape	1660	15.74	2896	6.73	206	29.22	272	24.22	343	23.24	5377	9.45
Limpopo	752	7.13	5757	13.37	10	1.42	44	3.92	40	2.17	6603	11.61
Eastern Cape	916	8.69	3596	8.35	49	6.95	52	4.63	90	6.10	4703	8.27
North West	736	6.98	3135	7.28	18	2.55	168	41.96	48	3.25	4105	7.22
Free State	713	6.76	4250	9.87	27	3.83	131	11.67	71	4.81	5192	9.13
Northern Cape	386	3.66	1203	2.79	2	0.28	41	3.65	16	1.08	1648	2.90
ex Swaziland	2	0.02	11	0.03	3	0.43	0	0.00	6	0.41	22	0.04
Foreign	2	0.02	2	0.00	0	0.00	0	0.00	3	0.20	7	0.01
Unknown	26	0.25	160	0.37	12	1.70	3	0.27	27	1.83	228	0.40

**Total**	**10 545**	**100.00**	**43 045**	**100.00**	**705**	**100.00**	**1123**	**100.00**	**1476**	**100.00**	**56 894**	**100.00**

*Source*: Health Professions Council of South Africa (HPCSA). HPCSA Statistics 2019. [cited 2021 Apr 15]. Available from: https://www.hpcsa.co.za/

AEA, Ambulance Emergency Assistant; BAA, Basic Ambulance Assistant; ECT, Emergency Care Technician; ANT, Paramedic; ECP, Emergency Care Practitioner.

### Age distribution

The mean age of registered emergency care personnel (at the time of registration) was 36 years (±8.8). As displayed in Table 3, almost half (42%) of the workforce was between 30 and 39 years old. The unknown categories of age were not included in this analysis.

**TABLE 3:** Distribution of emergency care personnel by age (*N* = 56 878).†

Category (in years)	AEA		BAA		ECP		ECT		ANT		Total	
	*N*	%	*n*	%	*N*	%	*n*	%	*n*	%	*n*	%
20–24	237	2.25	3646	8.47	27	3.83	14	1.25	5	0.34	3929	6.91
25–29	998	9.47	9083	21.10	146	20.71	149	13.27	75	5.09	10 451	18.37
30–34	1580	14.99	10 070	23.40	205	29.08	370	32.95	210	14.25	12 435	21.86
35–39	2127	20.18	8727	20.28	122	17.30	250	22.26	245	16.62	11 471	20.17
40–44	2024	19.21	5896	13.70	107	15.18	197	17.54	280	19.00	8504	14.95
45–49	1635	15.52	3348	7.78	69	9.79	109	9.71	317	21.51	5478	9.63
50–54	1102	10.46	1349	3.13	27	3.83	24	2.14	201	13.64	2703	4.75
55–59	580	5.50	615	1.43	1	0.14	9	0.80	97	6.58	1302	2.29
60–65	223	2.12	259	0.60	1	0.14	1	0.09	32	2.17	516	0.91
> 65	32	0.30	45	0.10	0	0.00	0	0.00	12	0.81	89	0.16

Source: Health Professions Council of South Africa (HPCSA). HPCSA Statistics 2019. [cited 2021 Apr 15]. Available from: https://www.hpcsa.co.za/

AEA, Ambulance Emergency Assistant; BAA, Basic Ambulance Assistant; ECT, Emergency Care Technician; ANT, Paramedic; ECP, Emergency Care Practitioner.

† , Excluding ‘unknown and under 20 years’ age category

### Population group

More than two-thirds (79.8%) of all personnel classified themselves as black people; 10.2% classified as white people and 5.6% as mixed race (Table 4).

**TABLE 4:** Distribution of emergency care personnel by population group (*N* = 56 894 [100%]).

Category	AEA		BAA		ECP		ECT		ANT		Total	
	*N*	%	*n*	%	*n*	%	*N*	%	*n*	%	*n*	%
White people	2315	21.95	2355	5.47	385	54.61	76	6.77	725	49.12	5856	10.29
Chinese	2	0.02	2	0.00	0	0.00	0	0.00	2	0.14	6	0.01
Indian	535	5.07	305	0.71	74	10.50	19	1.69	137	9.28	1070	1.88
Black people	6176	58.57	37 898	88.04	170	24.11	839	74.71	340	23.04	45 423	79.84
Mixed race	1158	10.98	1595	3.71	71	10.07	186	16.56	198	13.41	3208	5.64
Unknown	359	3.40	890	2.07	5	0.71	3	0.27	74	5.01	1331	2.34

**Total**	**10 545**	**100.00**	**43 045**	**100.00**	**705**	**100.00**	**1123**	**100.00**	**1476**	**100.00**	**56 894**	**100.00**

Source: Health Professions Council of South Africa (HPCSA). HPCSA Statistics 2019. [cited 2021 Apr 15]. Available from: https://www.hpcsa.co.za/

AEA, Ambulance Emergency Assistant; BAA, Basic Ambulance Assistant; ECT, Emergency Care Technician; ANT, Paramedic; ECP, Emergency Care Practitioner.

### Sex

Majority (55.0%) of the emergency care professionals were male. Similarly, in all five categories, male professionals were in the majority: 68.9% in AEA, 51.1% in BAA, 67.6% in ECP, 68.7% in ECT and 78.6% in ANT (see Table 5).

**TABLE 5:** Distribution of emergency care personnel by sex (*N* = 56 889 [100%]).

Category	AEA		BAA		ECP		ECT		ANT		Total	
	*N*	%	*n*	%	*n*	%	*N*	%	*N*	%	*n*	%
Male	7269	68.93	21 995	51.10	477	67.66	772	68.70	1161	78.66	31 674	55.67
Female	3276	31.07	21 045	48.90	228	32.34	351	31.20	315	21.34	25 215	44.32

Source: Health Professions Council of South Africa (HPCSA). HPCSA Statistics 2019. [cited 2021 Apr 15]. Available from: https://www.hpcsa.co.za/

AEA, Ambulance Emergency Assistant; BAA, Basic Ambulance Assistant; ECT, Emergency Care Technician; ANT, Paramedic; ECP, Emergency Care Practitioner.

### Forecasting the need for emergency care personnel

For 2020, it was calculated that there were 95.9 emergency care personnel per 100 000 population in South Africa.^29,30^

Thus, to keep the forecast more realistic, we forecasted the supply using the past registrations for the previous five years (2014, 2015, 2016, 2017, 2018). A negative trend in the growth was observed for registrations of four out of five categories, as registrations for the following four categories have been closed: BAA, AEA, ECT and ANT.

**FIGURE 2:**
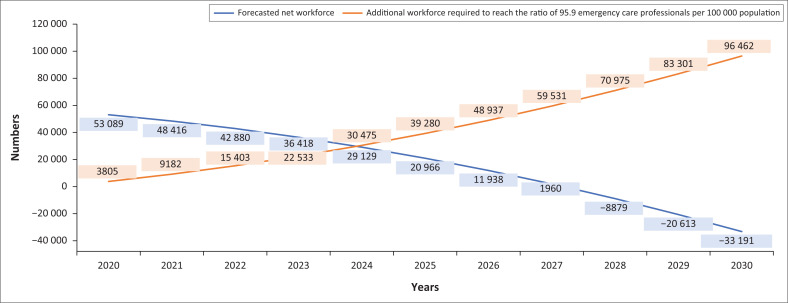
Forecasted need for emergency care personnel up to 2030 for South Africa for maintaining ratio of 95.9 emergency care personnel per 100 000 population.

It was observed that if the current trend continues and the registrations remain closed as on date, then in the year 2024, the net registered workforce will be less than the workforce needed to maintain the status quo of 95.9 emergency care personnel per 100 000 population (Figure 2). Thus, if this trend continues till 2030, an additional 96 000 personnel will be required to bring the emergency care registered workforce back to the current 95.9 emergency care personnel per 100 000 population ratio. However, it must be observed that the current number of actually employed EMS personnel is less than 50% of the emergency care personnel registered with HPCSA.

## Discussion

Emergency medical conditions generally occur through a sudden injury to the body or mind, often through injury, infection, obstetric complications or some chemical imbalance. They may also arise as a result of persistent neglect of chronic diseases. Emergency medical services treating these conditions constitutes rapid assessment, timely provision of right interventions and prompt transportation to the nearest health facility suitable for the patient by best possible means to increase chances of survival, control morbidity and prevent disability.^6^

This study sets out to describe the status of human resources for emergency care personnel in South Africa. The profile of an emergency care personnel employed in South Africa is likely to be black male and in the age group of 30–39 years, working in one of the economically better-resourced provinces. The distribution trends across three provinces (Gauteng, WC and KZN) over the last 15-year period show that they have always enjoyed more resources, and little has changed despite calls for a more equitable distribution of human resources. For instance, in 2019, Gauteng had 30.2% of emergency care personnel, KZN had 13.2%, Limpopo had 11.7% and WC had a 9.5% distribution of emergency care personnel.

Apart from registrations, it is also essential to see the provincial health expenditure by programme per province within South Africa. If we observe the health expenditure per province for 2019–2020, then Free State does maximum expenditure of 7.2% (of all provinces) on emergency health services, which is followed by Northern Cape (6.5%) and Eastern Cape (4.9%) (Table 6)^30^. Whereas national expenditure remains at 3.9% of the total health expenditure.^31^ In financial year 2020, emergency medicine was the fifth in terms of funding for national health expenditure, that is after District Health Services, Central Hospital Services, Provincial Hospital Services and Health Facilities Management.^31^

**TABLE 6:** Provincial health expenditure on emergency health services per province (Rand million), 2019–2020.

Province	SA	EC	FS	GP	KZ	LP	MP	NC	NW	WC
Emergency Health Services	8394	1278	808	1540	1603	818	419	337	436	1156
Total	21 5755	26 229	11 158	53 693	45 745	21 080	14 338	5223	12 488	25 802
Percent spending of total health expenditure (%)	3.9	4.9	7.2	2.9	3.5	3.9	2.9	6.5	3.5	4.5

*Source*: Day C, Gray A, Padayachee T, Cois A. Health and related indicators 2020. S Afr Health Rev. 2020;2020(1:264).

SA, South Africa; EC, Eastern Cape; FS, Free State; GP, Gauteng ; KZ, KwaZulu-Natal; LP, Limpopo; MP, Mpumalanga; NC, Northern Cape; NW, North West; WC, Western Cape.

The current ratio of emergency care professionals for South Africa is 95.9 per 100 000 population. However, as per an analysis undertaken for 50 most populous metropolitan areas in the United States, the top five areas had availability of Emergency Medical Technician (EMTs) and ANTs within the range of 102.7 to 121.3 per 100 000 population.^32^

It was observed that if the current trend continues and the registrations remain closed as on date, there will be a shortage of around 96 000 emergency care personnel by 2030. The net registered personnel will be short of 33 000 personnel and to maintain the current ratio of 95.9 emergency care personnel per 100 000 population, an additional 96 000 emergency care personnel will be needed. It is imperative that the current educational framework is revised and policy interventions are undertaken to avoid future shortages of trained emergency care personnel within South Africa. Also, it is unclear how many of these registered personnel (as per the HPCSA register) are active and contributing to the South African health sector. In a recent study, healthcare workers from South Africa responded having negative perceptions of the public health system because of high patient loads, long working hours, inadequate resources and occupational hazards, which in turn lead to either leaving or avoiding employment in the health sector.^33^ In addition, it has been reported that there is limited availability of employment positions within EMS.^34^

**FIGURE 3:**
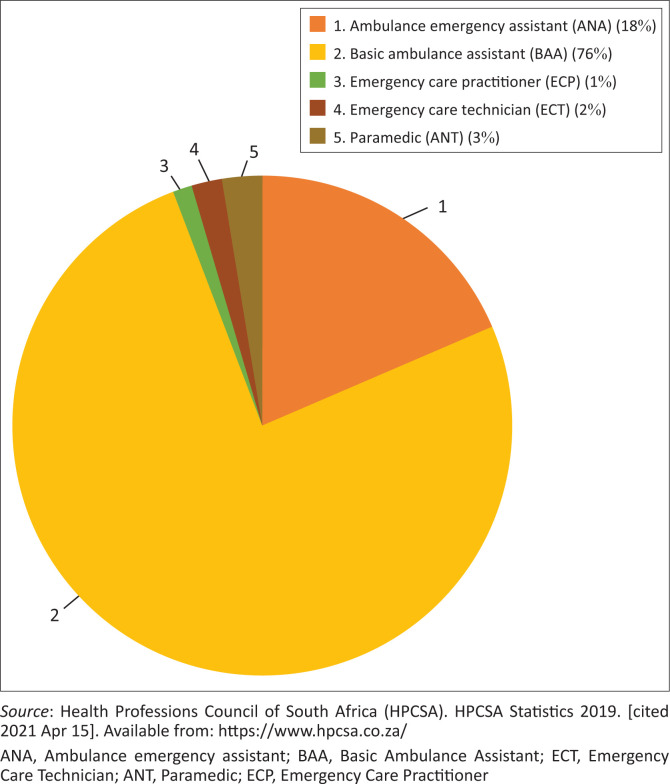
Current skills mix for emergency care personnel in South Africa (2019).

For South Africa, the current skills mix (Figure 3) represents a majority of BAA (76%), followed by AEA (18%), ANT (3%), ECT (2%) and ECP (1%). However, the ministerial appointed review committee recommended that the skills mix should have 45% EMS cadre at a basic life support level, 45% at intermediate and 10% at the advanced life support level. Thus, to reach the desired skills mix levels, the training and education system and licensing, professionalising and registration for emergency care personnel must be transformed. In 2017, the National Emergency Care Education and Training Policy already highlighted the need for enhancing and maintaining the quality of emergency care education.^19^

This study is timely and relevant as it provides a comprehensive and detailed summary of the current emergency care service delivery in terms of HRH within South Africa. Our findings highlight the prioritisation of resource utilisation in a resource deficit environment. This study can be used as a framework for other African countries and lower and middle-income countries to analyse current availability and estimation of optimum HRH levels of access to care for emergency care services.

The limitations of this study include that the accuracy of HPCSA figures cannot be fully guaranteed. The existing data only described the current situation. Most notably, this research only considers the number of personnel registered with HPCSA and not the number employed within EMS in South Africa. Some of the other key HRH aspects of the emergency care workforce are also unreported such as possible changes in the burden of disease, service delivery models, facility planning, facility workloads, skills mix, remuneration systems and health worker productivity, all of which are important in projecting future HRH needs and costs. The findings raise some concerning questions about the profession and specific implications for access to emergency care services. It should also be noticed that one of the author is involved with the revision of current educational framework thus there may exist a research bias in interpreting the policy data. Furthermore,, this study did not set out to assess if the syllabus or contents of any of the courses of Emergency Care Professionals was appropriate.

South Africa is working towards a National Health Insurance (NHI) system, thus for ensuring that the NHI has a significant transformative impact on the health sector related to emergency care services as well, we propose a two-pronged strategy to meet the inadequacy of emergency care professionals and to optimise their skills mix. Firstly, a step-by-step approach should be set up based on public sector colleges and higher education institutions and their training capacity. As a first step, public sector colleges should become HEIs for emergency care courses as provided by the Minister of Higher Education and Training (Notice 1040 of 2012; Government Gazette No. 36003 of 14 December 2012).^20^ The educational courses re-aligned as per suggested NQF levels should have ample seats to accommodate students with a target of meeting future workforce shortages. As a second step, the number of staff and infrastructural resources in public sector colleges should be scaled up. As a third step to not reduce the number of operational EMS personnel, service providers need to create or increase supernumerary positions (on a rotational basis).

## Conclusion

Under-resourcing and disparities in the profile and distribution of emergency care professionals remain an abiding concern that negatively impacts the provision of emergency care services and equitable health outcomes.
